# Effect of Fabrication Technology on the Accuracy of Surgical Guides for Dental-Implant Surgery

**DOI:** 10.3390/bioengineering10070875

**Published:** 2023-07-24

**Authors:** Lucio Lo Russo, Laura Guida, Pierluigi Mariani, Vincenzo Ronsivalle, Crescenzio Gallo, Marco Cicciù, Luigi Laino

**Affiliations:** 1 Department of Clinical and Experimental Medicine, School of Dentistry, University of Foggia, 71122 Foggia, Italy; 2 Salus Oris Srl, 83050 Vallesaccarda, Italy; 3 Multidisciplinary Department of Medical-Surgical and Odontostomatological Specialties, University of Campania, “Luigi Vanvitelli”, 81055 Naples, Italy; 4 Department of General Surgery and Medical-Surgical Specialties, School of Dentistry, Unit of Oral Surgery and Prosthodontics, University of Catania, 95124 Catania, Italy

**Keywords:** surgical guides, accuracy, milling, 3D printing

## Abstract

Background: The accuracy of surgical guides is a relevant factor in both surgical safety and prosthetic implications. The impact of widespread fabrication technologies (milling and 3D printing) was investigated. Methods: Surgical guides manufactured by means of two specific milling and 3D-printing systems were digitized and compared in a 3D analysis with the digital file of the designed guides. The surface mean 3D distance (at the surface where the teeth and mucosa made contact) and the axial and linear deviations of the sleeves’ housings were measured by means of a metrological software program. Univariate and multivariate statistical analyses were used to investigate the effects of the fabrication technology, type of support, and arch type on the surgical guides’ accuracy. Results: The median deviations of the intaglio surface in contact with the mucosa were significantly (*p* < 0.001) lower for the milled surgical guides (0.05 mm) than for the 3D-printed guides (−0.07 mm), in comparison with the reference STL file. The generalized estimated equation models showed that the axial deviations of the sleeves’ housings (a median of 0.82 degrees for the milling, and 1.37 degrees for the 3D printing) were significantly affected by the fabrication technology (*p* = 0.011) (the milling exhibited better results), the type of support (*p* < 0.001), and the combined effect of the fabrication technology and the sleeve-to-crest angle (*p* = 0.003). The linear deviation (medians of 0.12 mm for the milling and 0.21 mm for the 3D printing) of their center points was significantly affected by the type of support (*p* = 0.001), with the milling performing slightly better than the 3D printing. Conclusions: The magnitude of the difference might account for a limited clinical significance.

## 1. Introduction

Advancements in the application of digital imaging in dentistry and in computer-aided design (CAD)-software technologies make the virtual planning of dental-implant treatments possible. These treatments utilize cone-beam-computed-tomography data merged with surface-scan data to assess and define the optimal implant position for surgical safety [1] and favorable prosthetic support [2]. In fact, within the digital environment, anatomical structures, bone quality, and the residual ridge morphology can be easily analyzed, allowing the precise definition of the implant position in order to optimize surgical-risk management, as well as prosthetic functional and aesthetic outcomes. Once the virtual implant’s position is defined, this needs to be precisely transferred on the patient during the surgical procedure for the implant’s placement. Two main approaches have been described to transfer the planned information (the antero-posterior, vestibulo-lingual, and corono-apical positions, as well as the implant-axis angulation) into the surgical field and obtain the corresponding desired and planned implant position [2]. (i) The first is a dynamic system, based on a navigation device using an optical tracking system, which is capable of providing real-time information on the position of a surgical instrument in relation to a patient’s anatomy. This involves the use of digital technologies capable to capture the position of the surgical instruments and providing live feedback to the surgeon in order to reproduce the planned implant position. (ii) The second is a static system, which utilizes a surgical guide as a means to incorporate all the information for the mechanical guiding of surgical instruments to obtain the planned implant position. The static approach is used more frequently and substantiates the paradigm behind so-called computer-guided surgery [3]. All the information needed to reproduce the planned implant position during a surgical procedure for implant placement can be incorporated into a static surgical guide. In other words, an object (i.e., the surgical guide) is used that incorporates metal sleeves or sleeve-less housings where the surgical instruments are inserted and limited in their degree of freedom; in this way, the required orientation of surgical instruments within the sleeves can accurately reproduce the planned implant’s position and, at the same time, reduce the effect of the operator’s skills. The surgical guide and the corresponding sleeves offer mechanical guidance for surgical instruments, which increases the predictability and accuracy of the implant position in comparison to free-hand implant placement [4,5], may decrease surgical invasiveness, and improves patient comfort [6]. Nonetheless, there may be differences between the actual implant position (i.e., the position obtained after the implant’s placement) and the planned position [7]. This is a sensitive issue from a clinical point of view, with associated surgical and prosthetic implications; such an “error” has been often investigated as a measure of the accuracy of surgical guides [8,9,10]. This could be misleading and requires caution because addressing such errors should be based on the understanding that they are the cumulative results of all the possible sources of error during all the stages of a guided implant-placement procedure [11], and the interactions between them; these sources of error include not only dimensional inaccuracies in surgical guides [12], but also all the possible errors resulting from data acquisition [13], management [14], merging [15], surgical-guide stabilization [16], and bone features [17].

Surgical guides play a major role in static computer-guided implant surgery; their dimensional accuracy deserves special attention as a prerequisite for successful and safe surgery. The most relevant factors affecting the contribution of surgical guides to the overall accuracy of computer-guided implant surgery are the intaglio surface (i.e., the internal surface of the surgical guide fitting the supporting teeth or mucosa) and the sleeves’ housings (i.e., the cylinder-shaped holes containing the sleeves). The accuracy of the intaglio surface is responsible for adequate adaptation to the supporting tissues and structures, whereas accuracy of the sleeves’ housings, where the guiding elements are inserted, can guarantee the congruence between the planned and obtainable implant axes, as well as the correct position in the apico-coronal direction. Both of these key factors in surgical guides need to be as close as possible to those of the CAD project regarding their shapes, axes, and overall position. Hence, the three-dimensional (3D) accuracy of the intaglio surface and sleeves’ housings are critical features in surgical guides, which may be affected by their manufacturing process [18]. This is a relevant issue, especially in light of the increasing diffusion of the in-office manufacturing of surgical guides, where both the chosen production technology and variations in procedures, hardware, and materials may have an impact.

Both additive [8] and subtractive [19] computer-aided manufacturing (CAM) technologies are available for surgical-guide fabrication. The corresponding accuracy has seldom been investigated in comparative studies [18,20,21], and there is no clear evidence regarding which processing technology (milling or 3D printing) can provide surgical guides with better accuracy. It should be noted that milling and 3D printing currently encompass a wide range of technologies, systems, and equipment, each with its own features, advantages, and drawbacks, whose detailed description is beyond the scope of the present study. It has also been reported that modifications are still possible in the post-manufacturing stage [22,23], due to the post-processing [24] or dimensional stability intrinsic to the material [25]. Cost-effectiveness considerations in the use of different materials and technologies have also been reported [26]. For these reasons, a careful consideration of the specific clinical and laboratory settings, as well as the degree of manufacturing-protocol standardization, is required, and the provision of data regarding the effectiveness and accuracy of all the technologies/protocol combinations that can be used in surgical-guide manufacturing remains a clinically significant research issue.

In the present study, the accuracies of milled and 3D-printed surgical guides were investigated by means of a 3D digital analysis. The null hypothesis was that, for surgical guides fabricated either by milling or by 3D-printing, no differences would be found in the accuracies of the intaglio surfaces, nor in the axes or center points of the sleeves’ housings (at their entrances).

## 2. Materials and Methods

**Figure 1:**
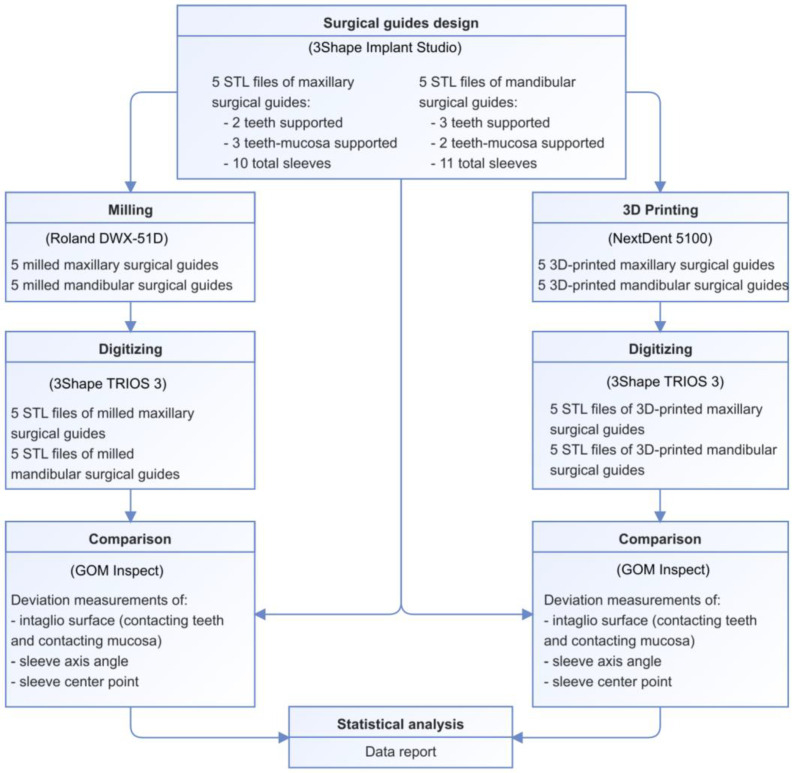
Overview of the study methodology.

An overview of the study’s methodology is presented in Figure 1. In a manner that was consistent with previous studies [18,21], the investigated sample size of 10 surgical guides (Table 1) was able to provide 80% power to detect a significant difference.

**Figure 2:**
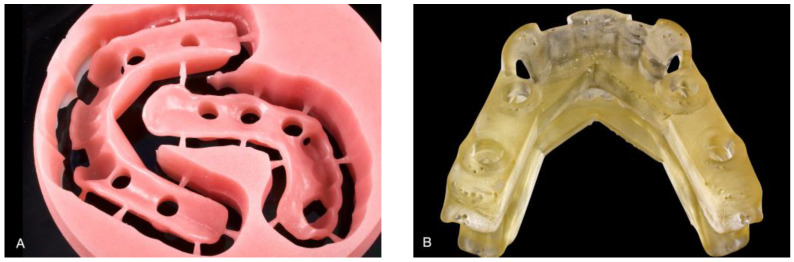
Examples of surgical guides: (**A**) milled surgical guides; (**B**) 3D-printed surgical guide.

All surgical guides were designed on intraoral scans retrieved from archives. Intraoral scans have been demonstrated to be sufficiently accurate, even when edentulous regions are present in the mouth [27]. The design of the surgical guides was performed by using a CAD software program (Implant Studio; version 2022.1; 3Shape A/S, Copenhagen, Denmark), which also generated the CAD output of the surgical guides in standard tessellation language (STL) files. Starting from each STL file, a milled and a 3D-printed surgical guide were fabricated (Figure 2).

For milled surgical guides, the STL file was used to create a CAM project in a polymethylmethacrylate blank with a height of 25 mm (Smile Cam Total Prosthesis; Pressing Dental Srl, San Marino, San Marino) by means of a software program (hyperDENT; FOLLOW-ME! Technology Group, München, Germany); milling was performed in a 5-axis milling machine (DWX-51D; Roland DG Corp, Hamamatsu, Japan). No further processing was performed after milling. A 3D printer (NextDent 5100; NextDent B.V., Soesterberg, The Netherlands) and the corresponding resin (NextDent SG; NextDent B.V., Soesterberg, The Netherlands) were used for 3D-printed surgical guides. According to the manufacturing protocol recommended by the manufacturer, before printing, the resin in the original packaging was shaken for 5 min by means of a roller-bench resin mixer. All surgical guides were fabricated from the same resin bottle. While printing, the surgical guides were oriented horizontally on the print platform, with the intaglio surface positioned opposite. A layer thickness of 0.05 mm was used, with support struts automatically generated by the 3D-printer software program; support struts were checked to ensure they did not cause an obstruction to the sleeves’ housings. Once printed, a putty knife was used to separate the surgical guides from the build platform. Next, according to the manufacturer’s instructions, surgical guides were ultrasonically cleaned in an alcohol solution (96%), dried, and placed in an ultraviolet-light unit (LC-3DPrint Box; NextDent B.V., Soesterberg, The Netherlands) for 10 min for postpolymerization. After this post-processing step, the support structures were removed. An intraoral scanner (TRIOS; 3Shape A/S, Copenhagen, Denmark) was used to scan each milled and 3D-printed surgical guide; scans were performed by one investigator, with special attention to the intaglio surface and sleeves’ housings. The selected intraoral-scanner technology has been demonstrated to provide reliable scans of both intraoral tissues (with accuracy demonstrated on both dentate and edentulous patients [27]) and extraoral models [28]. In particular, the scanning precision of the Trios 3 has been reported to be, on average, 49 μm [29]. The 3D-printed surgical guides were lightly coated with antiglare spray (Helling 3D Scan Spray; CyberOptics Corp, Minneapolis, MN, USA) with an average particle size, according to the manufacturer, of 2.8 µm.

**Figure 3:**
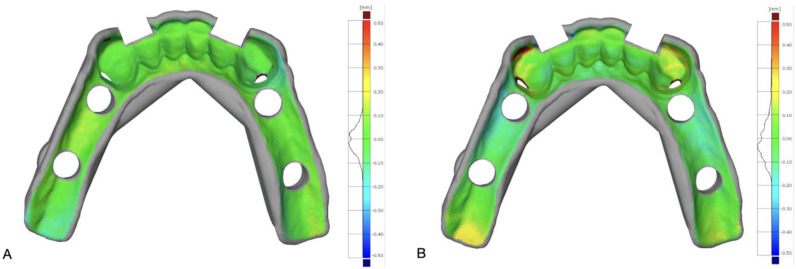
Intaglio surface of a surgical guide. Positive deviation on the color-deviation map (yellow to red) indicates tissue impingement. Negative deviations are shown by cyan-to-blue colors: (**A**) Milled; (**B**) 3D-printed.

The scans of the milled (M) and 3D-printed (S) surgical guides were exported as STL files and used for 3D analysis and comparisons in a metrological software program (GOM Inspect suite; Carl Zeiss GOM Metrology GmbH, Braunschweig, Germany). In particular, the STL files of M and S were compared with the STL file of the designed surgical guide (R), which was used as reference; to this end, M and R, as well as S and R, were aligned with each other. The intaglio surface of the surgical guide was used to optimize the superimposition by means of a local best fit. Mean 3D distance between M and R, as well as between S and R, was measured for the intaglio surface in contact with teeth, as well as for the intaglio surface contacting the mucosa. Because of the comparison schema described above, the trueness of intaglio surfaces of investigated surgical guides were measured at both surfaces in contact with teeth and mucosa. A color-difference map of each comparison was used for visual analysis (Figure 3).

**Figure 4:**
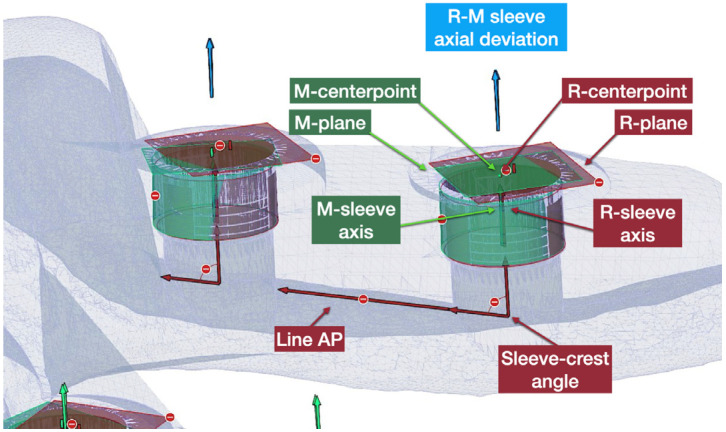
Diagram of the methodology used for measuring axial and linear deviations of sleeves’ housings. M: features (cylinders, planes, and centerpoints) built into the STL file of the scan of the milled surgical guide. R: features (cylinders, planes, and centerpoints) built into STL file of the designed (CAD) surgical guide. Blue arrows: M and R cylinder axes. The sleeve to crest angle is defined by the intersection between cylinders’ axis and Line AP.

The methodology described by Lo Russo et al. [30] (Figure 4) was used to measure axial and linear deviations of sleeves’ housings.

Briefly, for each sleeve, the axis of the corresponding cylindrical housing was determined by automatically creating and fitting a cylinder to it. A reference plane was built at the entrance of each sleeve: the intersection of this plane with the previously identified axis allowed us to identify the center point at the sleeve’s entrance. This procedure was performed on R, M, and S; next, for the R–M and R–S pairs, the axial and linear deviations of sleeves’ housings were calculated. The former (axial deviation) was defined as the angle between sleeves’ housings axes on superimposed R–M and R–S pairs; the latter (linear deviation) was defined as the distance between center points at the entrances of sleeves’ housings on superimposed R–M and R–S pairs. An arbitrary mesiodistal line was also constructed on the intaglio surface of the surgical guide on the edentulous ridge crest and used to calculate the angle between the axis of sleeves’ housings and the ridge profile. This was because angulation of the sleeve in relation to the body of the surgical guide may impair access for the milling tool; similarly, maintaining the position of the surgical guide’s body on the 3D-printing platform, the angulation of the sleeve may affect the quality of the finishing of the sleeves’ housings and their corresponding accuracy.

The mean values of the analyzed surface deviations were assessed for differences between groups by means of the Mann–Whitney test. The mean 3D deviations in surface comparisons (R–M: milled versus designed surgical guides; S–R: 3D-printed versus designed surgical guides) were also tested for statistical significance by testing the following hypothesis: the distance between the compared surfaces is zero if they are identical. Hence, the one-sample *t* test was used to answer the following question: Is the observed mean distance significantly different from zero?

The effects of manufacturing technology between and within surgical guides on discrepancies for axial and linear deviations of sleeves’ housings were also investigated by using two generalized estimated equation (GEE) models, in order to model and control the within-unit measurements. Axial and linear deviations of sleeves’ housings were individually investigated as dependent variables in the GEE models. For each of them, the surgical guide was used as subject variable. Fabrication technology (milling/3D printing), the type of support in surgical guides (tooth-supported/mixed-supported), and arch type (maxillary/mandibular) were used as factors. The angle between the sleeve axis and the ridge profile was included as a covariate. In the model addressing axial deviation, the linear deviation was also used as covariate, and vice versa. Additionally, the combined effects of independent variables were considered in the models. All statistical analyses (α = 0.05) were performed by using a statistical software program (IBM SPSS Statistics, v25.0; IBM Corp, New York, NY, USA).

## 3. Results

The investigated surgical guides and their features are detailed in Table 1. A total of ten surgical guides (five for the mandibular arch and five for the maxillary arch, with twenty-one sleeves in total), fabricated with additive and subtractive digital technologies, were analyzed. Their accuracies (the median difference between the milled or the 3D-printed surgical guide and the designed surgical guide used as a reference) are summarized in Table 2 and Table 3.

In particular, in Table 2, the surface accuracy is reported, as a distinct measurement at the intaglio surface in contacting with the teeth or mucosa. This distinction was made because, according to the types of tissue supporting the surgical guide, the 3D shape or the intaglio surface was sensibly different, thus offering a different level of manufacturing difficulty, which may have affected the corresponding accuracy. In fact, the surfaces contact with the teeth showed, on average, for both the milling and the 3D printing, higher accuracy with less variation. The median accuracy of the surface in contact with the teeth was 0.00 mm for both the milling and the 3D printing, with an interquartile-range value of 0.01 mm, whereas the median accuracies of the surface in contact with the mucosa were 0.05 mm and −0.07 mm (with interquartile-range values of 0.07 mm and 0.12 mm) for the milling and 3D printing, respectively.

In Table 3, the data regarding the sleeves’ housings’ axial deviations, as well as the linear deviations of the center points at the sleeves’ housings entrances are detailed. The milling exhibited slightly better accuracy regarding the sleeves’ housings’ axial deviations. In fact, a median axial deviation of 0.82 degrees (interquartile range, 1.11 degrees) was found for the milled surgical guides, whereas it was 1.37 degrees for the 3D-printed surgical guides (interquartile range, 1.00 degrees). The same trend was obtained for the linear deviations of the center points at the sleeves’ housings entrances. A median of 0.12 mm (interquartile range, 0.03 mm) was measured for the milled surgical guides, and a median of 0.21 mm (interquartile range, 0.12 mm) was obtained for the 3D-printed surgical guides.

Regarding the surface accuracy, the measured surface deviations were significantly different from zero (one-sample Wilcoxon rank test) only for the intaglio surface in contact with the mucosa (median: 0.05 mm, *p* = 0.014; median: −0.07 mm, *p* = 0.012 for the milled and 3D-printed surgical guides, respectively). The axial (medians of 0.82 degrees for the milling and 1.37 degrees for the 3D printing) and linear (medians of 0.12 mm for the milling and 0.21 mm for the 3D printing) deviations of the sleeves’ housings (Table 3) were significantly different from zero (*p* < 0.001), regardless of the manufacturing technology.

The GEE models fitted to the deviations of the sleeves’ housings showed that the axial deviations were significantly affected by the fabrication technology (*p* = 0.011), the type of surgical-guide support (*p* < 0.001), and the combined effect of the fabrication technology and the sleeve-to-crest angle (*p* = 0.003); for all these factors, the milling showed a slightly better performance than the 3D printing. The linear deviation of the center points (at the entrances) of the sleeves’ housings was significantly affected by the type of support for the surgical guides (*p* = 0.001).

## 4. Discussion

The null hypothesis (i.e., the absence of an effect of the manufacturing technology on the investigated parameters related to the sleeves’ housings) was partially retained because only the trueness of the intaglio surface in contact with the teeth was not affected by the manufacturing technology. By contrast, some deviation from the reference CAD model was found for the other investigated parameters in both the milled and the 3D-printed surgical guides: for the remaining parameters (i.e., the deviation of the surface in contact with the mucosa, and the sleeves’ housings’ axial and linear deviations), the null hypothesis was rejected.

While the deviations in the surfaces in contact with the teeth were generally the same for the milled and 3D-printed surgical guides, the milling showed, on average, higher accuracy with less variation on the surfaces of the surgical guides in contact with the mucosa (0.05 mm for the milling and −0.07 mm for the 3D printing); although the magnitude of this difference was very small, it is worth noting that it was opposite in direction. The better performance of the milling in obtaining a higher accuracy for the intaglio surfaces of the surgical guides in contact with the mucosa confirmed the findings in recent reports on complete dentures.

The intaglio-surface accuracy in the present study was higher than in a previous study [18] (0.21 mm for the milling and 0.23 mm for the 3D printing), in which no differences between the internal surfaces in contact with the teeth or the mucosa were found. Axial deviations in the sleeves’ housings have not been reported so far in other studies with comparable methodologies, involving the use of the CAD project of the surgical guide as a reference for the outcome measurements, and investigating milling and 3D printing at the same time. Data regarding the linear deviation of the center points (at the entrances) of sleeves’ housings have been reported instead [18]. Although, in [18], the linear deviation was differentiated in the horizontal and vertical directions, and between the anterior and posterior zones, the magnitude (ranging from 0.11 mm to 0.25 mm for the milling, and from 0.22 mm to 0.4 mm for the 3D printing) was similar to that in the present study (0.12 mm for the milling and 0.21 mm for the 3Dprinting).

The results in the present study showed that the milling and 3D printing produced surgical guides with different accuracies. Nonetheless, some of the measured deviations from the reference value, especially the axial and linear deviations of the sleeves’ housings, were significantly different from zero (*p* < 0.001), regardless of the manufacturing technology. Hence, the ideal goal of a surgical guide with zero deviation from its reference CAD project, seems unreachable because of the tolerance inherent in each manufacturing methodology. In other words, it seems necessary to accept some inaccuracy in the surgical guide, due to the intrinsic tolerance of specific manufacturing processes. As a consequence, it would be sensible to estimate the potential impact of the measured fabrication inaccuracy on the final implant-position error, in order to define, statistical significance apart, the potential clinical significance. This is quite straightforward with a recently described technique [30], based on the understanding that the final implant position can also be affected by several sources of error unrelated to the surgical guide itself; thus, the precise estimation of actual implant errors is not feasible with the validation of the surgical guide after manufacturing alone. Based on the findings in this study, assuming a fully guided surgical system with a 9-millimeter sleeve offset (the distance of the sleeve entrance from the implant platform), and with a 10-millimeter implant length, the surgical-guide inaccuracy resulting from the milling and 3D-printing manufacturing processes (0.82 and 1.37 degrees of axial deviation, respectively) can cause [30] about 0.3–0.5 mm of lateral error at the implant apex, as well as 0.1–0.2 mm of lateral error at the implant platform. Such errors need to be considered together with the measured linear deviations (0.12 and 0.21 mm, respectively). Thus, in the suggested situation (9-millimeter sleeve offset, 10-millimeter implant length), a 3D-printed surgical guide, compared to a milled guide, might yield 0.2 mm of additional lateral error at both the implant apex and the implant platform. Considering that in many implant-position-planning software programs, a safety zone of 2 mm around implants is generally taken into account to validate the project, the reported errors, as well as the differential errors caused by the 3D-printing-based fabrication of a surgical guide, might be of limited clinical relevance. Nonetheless, it must be noted that by modifying sleeve offset and implant length, the calculation errors may be significantly changed. Thus, a careful evaluation, on a case-by-case basis, of all the relevant variables, including surgical-guide-fabrication technology, is mandatory, bearing in mind that surgical-guide accuracy may be responsible for only a fraction of the cumulative final errors in the implant position; thus, it remains for the clinician/surgeon to take care of all the other sources of potential errors.

The results in this study are applicable to the specific experimental settings, equipment, and materials used. This could be a potential limitation; therefore, further clinical and experimental studies need to be conducted, and these should include other additive-manufacturing technologies, materials combinations, and measuring devices. Regarding the latter, the reported 0.049-millimeter precision [29] of the scanner used in the present study should be considered, although the comparative study design (i.e., the measurement of the difference between two similar objects using the same instrument) may minimize the effect of the reported results.

In our study, we did not measure the absolute dimensions of the surgical guides; thus, regardless of the nature of the instrument error, in this repeated-measurement design, it has little, if any, relevance to the measured difference.

## 5. Conclusions

Based on the findings of the present study, the following conclusions were drawn:

1. The fabrication technology of surgical guides may affect their accuracy for the surface in contact with the mucosa, as well as the axial accuracy of the sleeves’ housings and their center points’ linear deviations.
2. For the parameters above, the milling showed statistically significantly better accuracy.
3. Considering the small magnitude of the measured differences, their clinical significance might be limited, although further investigations are required, including all the possible combinations of manufacturing technologies and their corresponding materials.

## Figures and Tables

**Table 1:** Surgical guides included in the study and their features.

	Maxillary Arch	Mandibular Arch	Total Dataset
Number of surgical guides	5	5	10
Number of sleeves	10	11	21
Sleeve-to-crest angle (degrees; mean ± standard deviation)	88 ± 8.3	80.9 ± 6.9	84.3 ± 8.3

**Table 2:** Surface deviations (trueness) of investigated surgical guides from their computer-aided design (CAD) reference files.

Surface Deviation	Fabrication Technology							
	Milling				3D Printing			
	Median	25th Percentile	75th Percentile	IQR *	Median	25th Percentile	75th Percentile	IQR *
Deviation of surface in contact with teeth (mm)	0.00	0.00	0.00	0.01	0.00	0.00	0.00	0.01
Deviation of surface in contact with mucosa (mm)	0.05	0.01	0.09	0.07	−0.07	−0.15	−0.03	0.12

* interquartile range.

**Table 3:** Axial and linear deviations of sleeves’ housings.

Deviation of Sleeves’ Housings	Fabrication Technology							
	Milling				3D Printing			
	Median	25th Percentile	75th Percentile	IQR *	Median	25th Percentile	75th Percentile	IQR *
Axial deviation (degrees)	0.82	0.32	1.43	1.11	1.37	0.71	1.71	1.00
Linear deviation of center points at sleeve entrance (mm)	0.12	0.10	0.13	0.03	0.21	0.12	0.24	0.12

* interquartile range.

## Data Availability

Data sharing is not applicable to this article.
